# Hydroxytyrosol: The Phytochemical Responsible for Bioactivity of Traditionally used Olive Pits

**DOI:** 10.5005/jp-journals-10018-1278

**Published:** 2019-02-01

**Authors:** Rengin Reis, Hande Sipahi, Gülşah Zeybekoğlu, Nur Çelik, Hasan Kırmızıbekmez, Neşe Kaklıkkaya, Ahmet Aydın

**Affiliations:** 1 Department of Toxicology, Yeditepe University, Istanbul, Turkey; 2 Department of Toxicology, Karadeniz Technical University, Trabzon, Turkey; 3 Department of Pharmacognosy, Yeditepe University, Istanbul, Turkey; 4 Department of Microbiology, Karadeniz Technical University, Trabzon, Turkey

**Keywords:** Analgesic, Anti-inflammatory, Hydroxytyrosol, Nitric oxide, *Olea europaea*, Olive pit.

## Abstract

The fruits of *Olea europaea* L. is widely consumed as food, and olive pits are utilized in folk medicine to relieve gastric disturbances. In the present study, the possible anti-inflammatory, analgesic and antioxidant activities of aqueous extracts of black (BP) and green olive (GP) pit prepared at gastric fed state pH were evaluated *in vitro.* Moreover, the bioactive compound, hydroxytyrosol (HT), was isolated from the extracts for the first time. According to results, GP extract (62.5 to 1000 μg/mL) showed significant anti-inflammatory activity in a dose-dependent manner and HT displayed significant nitrite inhibition at 100 μM with slight analgesic activity. Extracts and HT showed a significant antioxidant activity according to Total Antioxidant Capacity (TOAC), cupric ion reducing antioxidant capacity (CUPRAC), and 2,2-diphenyl-1-picrylhydrazyl (DPPH) radical scavenging assays. As a conclusion, a proper formulation containing HT might be a potential remedy to relieve gastric disturbances and olive pits, can be utilized as a valuable industrial tool for the low-cost production of HT.

**How to cite this article:** Reis R, Sipahi H, Zeybekoglu G, Celik N, Kirmizibekmez H, Kaklikkaya N, Aydin A. Hydroxytyrosol: The Factor Responsible for Bioactivity of Traditionally used Olive Pits. Euroasian J Hepatogastroenterol, 2018;8(2):126-132.

## INTRODUCTION

Olive, known as *Olea europaea L.,* is the most popular member of the Olea genus. Moreover, it is the only species of the *Oleaceae* family that is consumed as a food.^1^ Particularly, olive is found in the Mediterranean region and consumed commonly in the Eastern Mediterranean Basin as well as Southeastern Europe, Northern Iran, Western Asia, and Northern Africa.^1^ Turkey has also an important potential for olive cultivation because of its geographic location and climate.^2^ According to International Olive Council (IOC) report (2015), over the last 25 years, the growth of olive consumption has been the strongest among the non-European Union members, especially in Turkey and Morocco.^3^ Besides its culinary importance, olive is also studied for its therapeutic effects. Indeed, there are many studies investigating the antioxidant, antimicrobial, anti-inflammatory, antidiabetic, laxative, and anticancer properties of the fruit itself^4^ or its derivatives such as its leafs,^1^ or olive oil,^5^ given its phenolic antioxidant content that has been related to the beneficial effects of Mediterranean diet^4,6^ or as a protector against the development and progression of inflammatory diseases.^6^ Over the last decade, ingestion of olive pits to relieve the symptoms of duodenal ulcer and gastric disturbances has become popular in Turkey following paramedical suggestions.^7^ However, ingestion of olive pits may lead to unwanted adverse effects on the gastrointestinal system due to the shape and the indigestible structure of the olive pit.^8^ According to a case report from Bulgaria, a patient had totally obstructed his pyloric channel after having swallowed several olive pits to cure his peptic ulcer following an ancient Bulgarian belief.^7^ In another case report,^8^ distal pyloric stenosis perforation and gastric phytobezoar were observed due to excessive olive pit ingestion.

To the best of our knowledge, there is no study that examines the biological activity of olive pits treated at gastric pH even though ingesting them is a traditional medical practice in many cultures. This study is the first to examine the potential effects of olive pits extracts prepared at gastric fed state pH, which sets a model to enlighten their effect when swallowed. In this study, we aimed to identify the possible anti-inflammatory, analgesic, antimicrobial, and antioxidant activities of aqueous extracts of black and green olive pits *in vitro.* Also, the isolation of the main bioactive compound was achieved and the same activity studies were performed for this compound as well.

## MATERIAL AND METHODS

### Chemicals, Reagents and Equipment

*Thin layer chromatography (TLC):* SiO_2_ plates (silica gel 60 *F_254_; Merck)* aluminum plates; eluents CH_2_Cl_2_-MeOH-H_2_O (80:20:2), visualization by spraying with 1% vanillin/ H_2_SO_4_ reagent followed by heating at 105^o^C for 2 to 3 min. Medium-pressure liquid chromatography (MPLC): Sepa-core^®^ Flash Systems X10/ X50 (Buchi),Redi sep columns (LiChroprep C_18_, 50 g, Teledyne Isco). Sodium phosphate monobasic, copper sulfate and ammonium molybdate were from Riedel-de Haen (Germany). Sulfuric acid, DPPH (2,2-diphenyl-1-picrylhydrazyl), ascorbic acid, LPS (lipopolysaccharide from *E.coli* 0111:B4), “N-Nitro-L-argi-nine methyl ester hydrochloride”., sulfanilamide, MTT (3-(4,5-dimethylthiazol-2-yl)-2,5-diphenyltetrazolium and N-(1-naphthyl) ethylenediamine dihydrochloride were obtained from Sigma Aldrich (USA). Butylated hydroxytoluene (BHT) was purchased from Doğa Drug Company (Turkey). Phosphoric acid was from Mettler (Switzerland). Neocuproine was obtained from Santa-Cruz Biotechnology (USA) and ammonium acetate was from Merck (Germany). Indomethacin and sodium nitrite were purchased from Fluka Chemika (Germany). For the cell culture, dulbecco’s modified eagle’s medium (DMEM) from Gibco (England) and fetal bovine serum (FBS), streptomycin and penicillin were used from Gibco (USA). Prostaglandin E2 Enzyme-linked immunosorbent assay (ELISA) Kit was purchased from Abcam (UK). UV-spectrophotometric plate reader was used from Thermo Multiskan Spectrum (Finland).

### Plant Material

The fruits of *Olea europaea L.* (Marmarabirlik) were purchased from a local market in Turkey. The representatives of samples are being kept in our laboratory.

### Preparation of Extracts

**Fig. 1:**
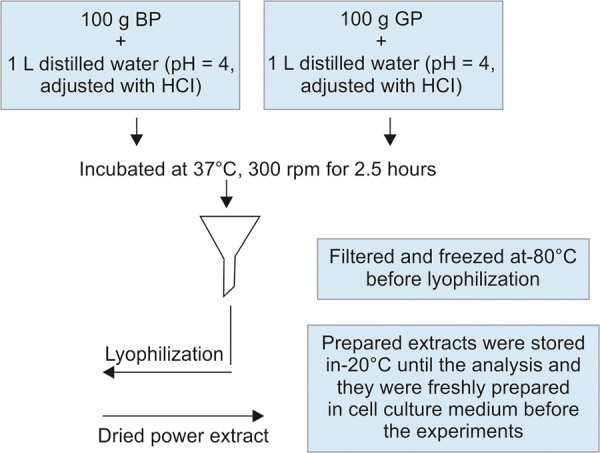
Schematic diagram of olive pit extraction and sample preparation processes. BP: Black olive pit extract; GP: Green olive pit extract

100 g of black olive pits (BP) (135 pits) and green olive pits (GP) (113 pits) were extracted separately by using 1 L of distilled water which was adjusted to pH 4 with HCl to simulate the fed state of gastric environment at 37^o^ C and then slightly shaken at 300 rpm for 2.5 hours, which is approximate time for gastric emptying.^9,10^ After the extraction process, the aqueous extracts were lyophilized and kept in -20^o^ C till use. Figure 1 shows the schematic diagram of the extraction and sample preparation processes.

### Isolation and Structure Elucidation of Bioactive Compound

**Fig. 2:**
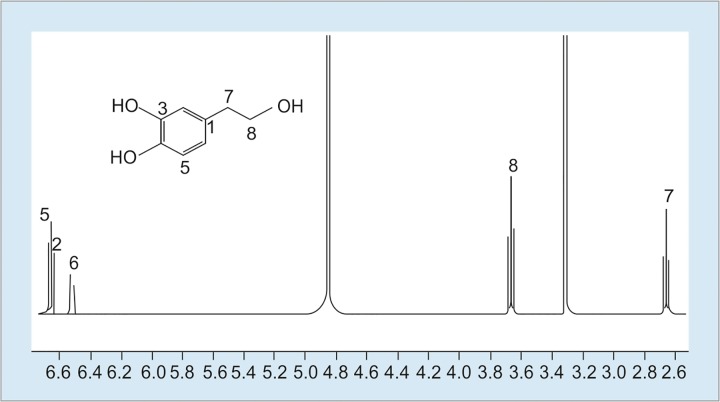
1H-NMR (400 MHz, CD3OD) Spectrum of hydroxytyrosol

**Fig. 3:**
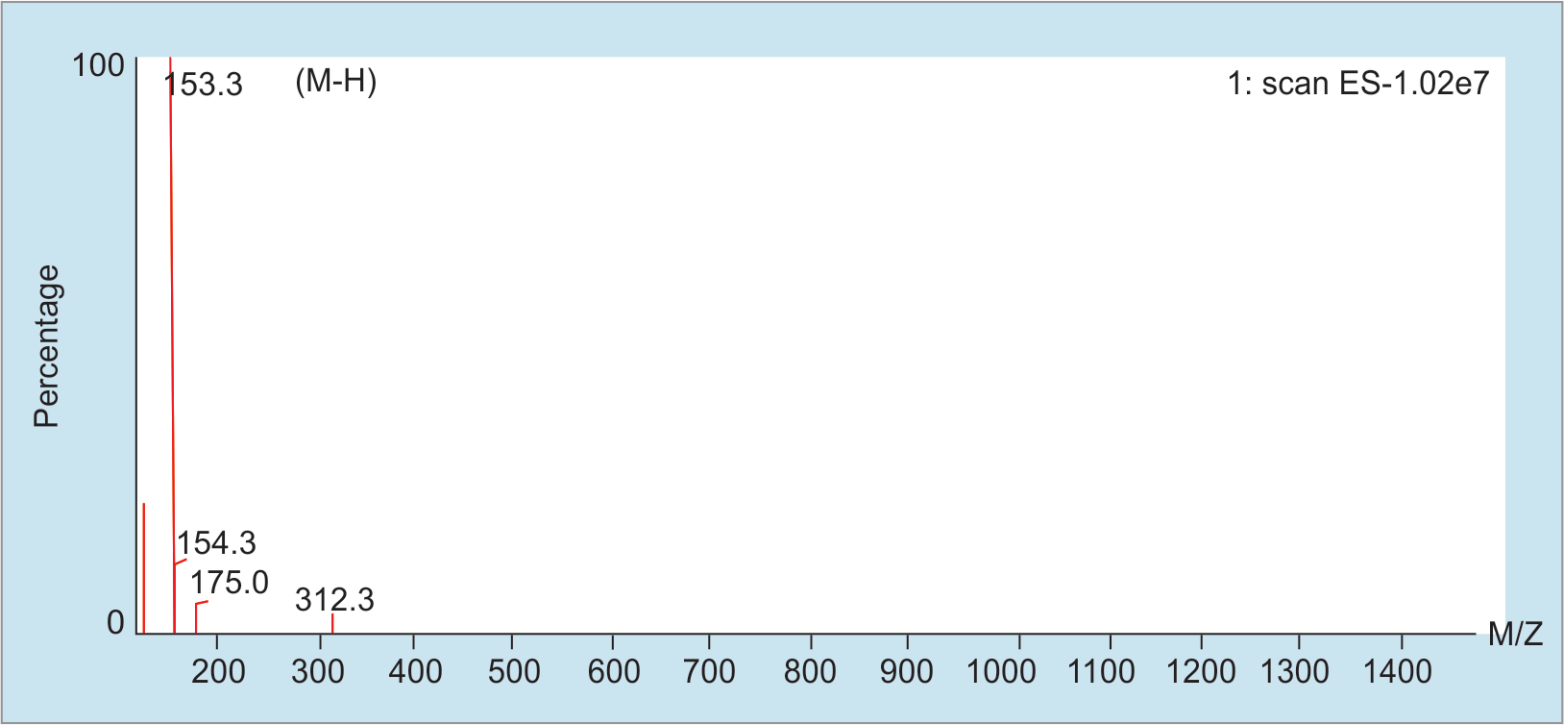
ESI-MS Spectrum of hydroxytyrosol.

The crude extract (green olive pit, 100 mg) was applied to Medium Pressure Liquid Chromatography (C_18_-MPLC, 50 g) eluting with stepwise gradient of MeOH in H_2_O (0-30% in steps of 5%) to yield pure hydroxytyrosol (HT, 3 mg). The structure elucidation was performed by using ^1^H NMR (400 MHz) and ESI-MS analysis (Figs 2 and 3).

### Cell Culture and Cell Viability

RAW264.7 murine macrophage cells (ATCC, USA) were maintained in DMEM, supplemented with 10% FBS and 1% streptomycin and penicillin at 37°C in 5% CO_2_. Cell viability was examined by using MTT assay. Plated RAW264.7 cells (10^6^ cells/ mL) were treated with different concentrations of olive pit extracts (62.5, 125, 250, 500 and 1000 μg/mL) and with the bioactive compound, HT (100μM). After 24 hours incubation process, MTT was added to each well at 0.5 mg/mL of concentration and incubated for an additional 2 hours at 37°C. After discarding all medium from plates, 100 μl of isopropanol was added to all wells. Absorbance of the blue formazan was determined at 570 nm by a UV-spectrophotometric plate reader (Thermo Multiskan Spectrum, Finland). Viability was defined as the ratio (expressed as a percentage) of absorbance of treated cells to untreated cells and all measurements were done in triplicates.

### Evaluation of Anti-inflammatory Activity

Anti-inflammatory activity of olive pit extracts was evaluated by measuring the stable nitric oxide (NO) metabolite, nitrite, with Griess reagent.^11^ Briefly, RAW264.7 cells were plated at the density of 10^6^ cell per mL in a 48 well-plate and incubated for 24 hours at 37°C in 5% CO_2_. Plated cells were pre-treated with five different concentrations of aqueous extract of olive pit (62.5, 125, 250, 500 and 1000 μg/mL) for 2 hours and then stimulated with 1 μg/mL of lipopolysaccharide (LPS) from *E. coli* 0111:B4, Sigma, USA) for additional 22 hours. The culture supernatant (50 μL) was mixed with Griess reagent [1% sulfanilamide and 0.1% N-(1-naphthyl)] ethylenediamine dihydrochloride in 5 % phosphoric acid] and incubated at room temperature for 10 minutes. The absorbance of the mixture was determined at 540 nm using a microplate reader (Multiskan Ascent, Finland). The amount of nitrite in the test samples was calculated by using sodium nitrite standard curve. 100 μM of indomethacin and 100 μM of L-NAME were used as positive control.

### Analgesic Activity

Analgesic activity was determined with Prostaglandin E2 ELISA Kit according to the manufacturer’s instruction by using cell supernatants of anti-inflammatory activity assay.

### Determination of Total Antioxidant Capacity

The total antioxidant capacities of samples were performed by applying the slightly modified version of the phosphomolybdenum method described by Celep et al.^12^ The assay was based on the reduction of Mo (VI) to Mo (V) by the antioxidant compounds and the subsequent formation of a green phosphate/Mo (V) complex at acidic pH. The reagent solution was consisted of 28 mM sodium phosphate monobasic, 4 mM ammonium molybdate and 0.6 M sulfuric acid. 0.03 mL of properly diluted samples was mixed with 0.3 mL reagent solution. The tubes containing the mixture were tightly capped and incubated at 95^o^ C for 90 minutes. After the incubation period, the samples were cooled to room temperature and the absorbance was read at 690 nm. The total antioxidant capacities were identified as mg ascorbic acid equivalent per g dried extract.

### Cupric Ion Reducing Antioxidant Capacity Assay

The cupric ion reducing capacities of the extracts were determined on 96-well plates as previously described.^13^ Briefly, 45 μl standard/sample and 50 μl distilled water were mixed. Subsequently, equal volumes of copper sulfate, neocuproine, and ammonium acetate were added. After 20 minutes of incubation period at 50^o^ C, the absor-bance was recorded at 450 nm. Ascorbic acid was used as standard and the results were expressed as mg ascorbic acid equivalent per g dried extract.

### DPPH Radical-scavenging Activity

DPPH radical-scavenging activity was performed on 96-well plates as previously described.^13^ Briefly, 50 microliter of each diluted extract was mixed with 250 microliter of freshly prepared 0.1 mM DPPH solution prepared in methanol. The mixture was vortexed and incubated in the dark for 50 minutes at room temperature. The absor-bance was measured at 517 nm. Butylated hydroxytoluene (BHT) was used as the reference substance while metha-nol was used as control. DPPH radical-scavenging activity was calculated as follows:

DPPH radical-scavenging activity (%)= [(Abs_control_-Abs_sample_) / Abs_control_] × 100

Abs_control_ is the absorbance value of the control group, and Abs_sample_ is the absorbance of the samples.

### Antimicrobial Activity

The extracts were screened against six bacterial strains *[Staphylococcus aureus* ATTC 25923 (gram-positive cocci), *Escherichia coli* ATTC 25922 (gram-negative rod), *Bacillus subtilis* ATTC 6633 (gram-positive rod), *Moraxella catarrha-lis* ATTC 25238 (gram-negative diplococci), *Lactobacillus casei* ATTC 27139 (gram-positive rod), *Helicobacter pylori* Sydney Strain-1 (microaerobic gram-negative rod)] and a fungal strain *(Candida albicans* ATTC 60193 (yeast)]. Antimicrobial activity tests were carried out by disk diffusion method as previously described.^14^ As reference substances, the following antimicrobials were used: Amoxicillin with clavulanic acid for *S. aureus* and *E. coli,* rifampin for *B. subtilis,* erythromycin for *L. casei,* fluco-nazole for *C. albicans,* and clarithromycin for *H. pylori.* Discs containing 20 μL DMEM (Gibco, England) were used as negative control.

## RESULTS AND DISCUSSION

It is well known that olive and its products have important nutritive and therapeutic effects due to their diverse phytochemical content.^1,15^ In the present study, we investigated the antioxidant, antimicrobial, anti-inflammatory, and analgesic activity of two types of marketed olive’s pits (green and black) by extracting them at gastric fed state pH (pH=4.0). The main reason for adjusting pH at 4.0 was to set a proper model for swallowed olive pits and to identify the main bioactive component responsible for the possible gastroprotective effects. Moreover, previous studies showed that the diffusion yield of phenolic compounds in different types of olive oils were greater at pH values higher than 2.0 (at pH 4.0 and 7.0), a datum compatible with our extraction pH selection for this study.^5^

**Fig. 4:**
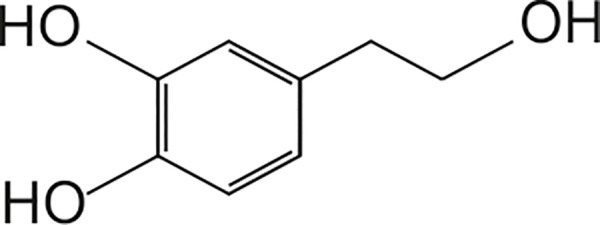
Chemical structure of hydroxytyrosol

The main bioactive component of olive pits at pH 4.0 was thought to possibly be HT since oleuropein, one of the well-known and most valuable phenolic compounds in olive, is described to be degraded to HT at gastric fed state pH.^1^ As a result of the chromatographic studies, the major chemical component of the extract was isolated. Its chemical structure was elucidated as hydroxytyrosol (HT) as seen in Figure 4 based on NMR and MS data and comparision of the findings with literature values.^16^

The olive pit extracts andHT did not show any cyto-toxic effect on RAW264.7 murine macrophage cells. The *in vitro* anti-inflammatory activities of the extracts and HT were assessed by nitrite assay, in which both indo-methacin (100 μM) and L-NAME (100 μM) were used as positive control. As seen in Table 1, GP extract exerted significant anti-inflammatory activity dose-dependently whereas, BP extract did not show any anti-inflammatory activity even at its highest dose (1000 μg/ mL). GP extract at 1000 μg/mL has nearly showed the same anti-inflammatory activity as bioactive compound HT at 100 μM. The anti-inflammatory effect of olive pits was investigated previously and 50 mg/L of extracted total polyphenols (mainly HT and oleuropein) were shown to reduce inflammation by decreasing NF-kB activity on human macrophage cell line.^17^ Herein, we demonstrated that GP extract showed significant anti-inflammatory activity; however, the same effect was not observed for BP extract in the same dose interval. GP (1000 μg/ mL) acted as the strongest anti-inflammatory substance when compared to BP (1000 μg/ mL). The most important finding of this study is that GP extract (500-1000 μg/mL) and HT (100 μM) exhibited considerably greater anti-inflammatory effect than indomethacin (100 μM) and the NO synthase inhibitor L-NAME (100 μM).

Additionally, the isolation of HT from olive pits is an important point of our study. Indeed, previous results showed that HT has an inhibitory effect on inflammatory mediators such as NO, PGE_2_, cytokines, interleukins, chemokines, etc.^18,19^ Moreover, Crea et al. (2012) suggested that the anti-inflammatory activity of HT may have an impact on neuroinflammation given its cytokine reducing effect in microglia cells. Furthermore, in the same study, the anti-inflammatory effect of HT on microglia was shown to be more effective than oleuropein, the other polyphenolic constituent of olive.^20^ Also, according to National Institute of Health (2017), currently there are three on-going clinical studies on HT. These studies are mainly indicated for the treatment/prevention of nonalcoholic fatty liver disease, breast cancer, and cardiovascular disease and are based on the anti-inflammatory, anti-atherogenic and antioxidant properties of HT. Other completed clinical trials on HT have demonstrated positive outcomes on inflammation, neuroinflammation and in the prevention of oxidative diseases when the compound was used as a dietary supplement.^21^ Thus, these findings suggest that olive pits, that are waste products, can be utilized as a valuable industrial tool for the low-cost production of bioactive HT.

**Table 1:** **Table 1:** Cell viability, nitrite inhibition and PGE_2_ levels.

*Groups*		*Doses*		*Cell viability %*		*Nitrite inhibition %%*		*PGE_2_ (pg/mL)*	
Control				100 ± 8.44		No inhibition		3437.09 ± 26.95	
		62.5 μg/mL		106.98 ± 7.82		11.30 ± 4.22		3400.43 ± 133.30	
		125 μg/mL		98.39 ± 5.97		19.58 ± 3.98 ***		3221.75 ± 277.57	
GP		250 μg/mL		110.86 ± 17.25		35.17 ± 6.80 ***		3343.25 ± 52.43	
		500 μg/mL		110.70 ± 10.39		70.63 ± 6.43 ***		3252.08 ± 76.50	
		1000 μg/mL		112.58 ± 12.61		89.68 ± 5.76 ***		2831.40 ± 88.80 **	
		62.5 μg/mL		105.25 ± 8.73		1.39 ± 6.49		-	
		125 μg/mL		94.86 ± 5.84		7.17 ± 4.21		-	
BP		250 μg/mL		91.40 ± 4.07		1.57 ± 3.37		-	
		500 μg/mL		87.35 ± 10.16		6.82 ± 3.34		-	
		1000 μg/mL		89.95 ± 5.79		8.47 ± 1.98		3418.24 ± 53.61	
L-NAME		100 μM		94.815 ± 7.56		42.73 ± 1.51 ***		3380.54 ± 53.02	
HT		100 μM		101.13 ± 2.47		84.78 ± 2.26 ***		2910.69 ± 68.47 *	
IND		100 μM		72.03 ± 10.81 **		49.98 ± 5.31 ***		449.31 ± 148.70 ***	

The significant differences between groups and control medium were defined with *p <0.05, **p <0.01 and ***p <0.001

BP: Black olive pit extract; GP: Green olive pit extract; LPS: Lipopolysaccharides from *E. Coli;* L-NAME: Nw-Nitro-L-arginine methyl ester hydrochloride; HT: Hydroxytyrosol; IND: Indomethacin.

Significant analgesic activity was observed in both GP extract (1000 μg/mL) and HT (100 μM) in PGE_2_ assay (Table 1). The analgesic activity of GP extract (1000 μg/ mL) was slightly higher than that of HT (100 μM). Indo-methacin (100 μM) was used as positive control in PGE_2 _assay and it showed greatest PGE_2_ inhibition. Due to the lack of anti-inflammatory activity of BP extracts in doses between 62.5- 500 μg/mL, only the highest experimental dose of BP (1000 μg/mL) was involved in PGE_2_ assay, which showed an insignificant inhibition. It is known that prostaglandins are involved in the maintenance of mucosal integrity^22,23^ and the protection of gastric mucosa against ulcerative challenge was related to the increase in prostaglandin content.^24^ Therefore, increase in gastric PGE_2_ content was correlated with the decrease in gastric lesion index and indomethacin, a nonspecific prostaglandin synthetase inhibitor, was found to reverse this protective effect by inhibiting PGE_2_ production.^24^ Thus, in this study, to follow the analgesic activity, PGE_2_ levels were monitored. GP (1000 μg/ mL) and HT (100 μM) reduced LPS-stimulated PGE_2_ production; however, the inhibition ratios of GP and HT were incomparably lower than indomethacin (100 μM). As their anti-inflammatory potency were higher than indomethacin (100 μM), the results suggest that the anti-inflammatory effect occurs with negligible PGE_2_ inhibition.

As shown in Figure 5, GP and BP extracts (62.5-1000 μg/mL) showed an increasing total antioxidant capacity in a dose dependent manner. GP has approximately 2.5 times higher antioxidant capacity than BP when both extracts were compared in higher experimental dose 1000 μg/mL.

The results were expressed as ascorbic acid equivalent (AAE) mg per g olive pit extract. Furthermore, the bioac-tive compound HT (100 μM) had significantly higher total antioxidant capacity compared to BP (all doses) and GP extract (except 1000 μg/mL).

The cupric ion reducing capacities of the extracts were represented in Figure 5. Cupric ion capacity of both extracts (62.5-1000 μg/mL) showed significant reducing capacity in a dose dependent manner. The highest activity was observed in 1000 μg/mL dose of GP extract. A greater reducing capacity was observed especially in doses higher than 250 μg/mL of GP compared to BP extract at the same dose interval. Moreover, the bioactive compound HT had significantly higher reducing capacity compared to BP (in all doses) and GP (in doses lower than 1000 μg/mL) extracts.

Both in the CUPRAC and TOAC assays, olive pit extracts showed an increasing antioxidant capacity in a dose dependent manner. These findings were supported by previously published data that demonstrated high antioxidant activity for both aqueous and ethanolic extracts of olive pit.^25^

**Figure:**
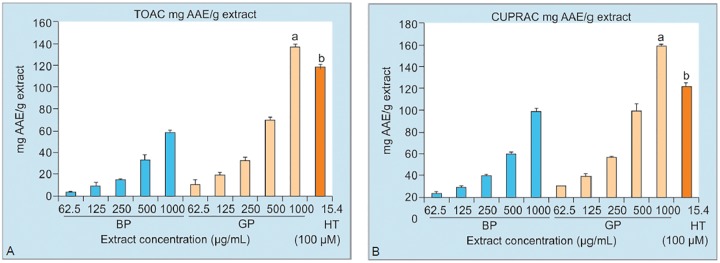
a: All groups vs. GP (1000 μg/ mL); b: HT vs. GP (1000 μg/ mL) statistically significant at p<0.001. CUPRAC: Cupric ion reducing capacity; BP: Black olive pit extract; GP: Green olive pit extract, and HT: Hydroxytyrosol **Figs 5A and B:** Total antioxidant capacity (A) and cupric ion reducing capacities (B) of HT (100 μM) and olive pit extracts (62.5-1000 μg/mL).

According to DPPH assay, the bioactive compound HT and GP showed higher free radical scavenging activity than BHT while BP exerted the weakest radical scavenging activity with an IC_50_ 580± 40 μg/mL. IC_50_ values of BP, GP, HT, and BHT were shown in Table 2. In radical-scavenging activity, the GP extract was more potent than the reference compound BHT while the BP extract was nearly 2.5 times less potent than the GP. As expected, HT (100 μM) was the most effective DPPH radical-scavenger with the lowest IC_50_ value.

Olive pit extracts prepared at their highest soluble concentration (BP and GP: 100 mg/mL and HT: 10 mM) did not significantly inhibit the bacteria or fungus strains tested. Polyphenols extracted from olive oil have been described for exhibiting anti*-H. pylori* activity and HT extracted from olive oil has been suggested to be bactericidal against lactic acid bacteria.^26^ However, in the present study, extracts and HT did not show any significant inhibition on the growth of neither of the tested strains in tested concentrations.

## CONCLUSION

Our study has shown that GP extract has shown strong antioxidant, anti-inflammatory, and analgesic activities especially in its highest experimental dose, 1000 μg/mL. Due to the lack of comparative data on the bioactivity of green and black olive pit in the literature, the difference in the content of the bioactive compound, HT, might be the reason for this notable difference. In many studies, the biological activities were mainly attributed to the total phenolic content of olive pits^4,6^ and it is known that the polyphenolic content and the profile of constituents in olives and their products may differentiate depending on cultivation, harvesting and processing types.^27^ Furthermore, it was reported that the phenolic composition of olives is negatively correlated with ripening.^15^ Therefore, differences in cultivation process and ripening degree between green and black olive might be another reason for this difference. It may be possible that GP extract is richer in HT in comparison to BP extract; therefore, it may show stronger activity in all experiments.

**Table 2:** **Table 2:** DPPH radical scavenging activities of BP, GP and HT.

		*BHT*		*GP*		*BP*		*HT*	
IC50 (μg/mL)		289 ± 7.0		210 ± 20		580 ± 40		9.27 ± 0.13	

BHT: Butylated hydroxytoluene (reference molecule), GP: Green olive pit extract, BP: Black olive pit extract, HT: Hydroxytyrosol

In conclusion, green olive pit extract had strong anti-inflammatory, antioxidant, and slight analgesic properties, which justify the traditional use of olive pits. These pharmacological activities are possibly related to its phenolic content, mainly HT. Thus, HT might be a potential therapeutic agent for the prevention and/or treatment of inflammatory diseases. However, it is worth to emphasize that excess amounts of olive pit should not be digested because of gastrointestinal perforation risk. Therefore, a proper formulation of olive pit extract and main bioactive compound HT might be a potential remedy to relieve gastric disturbances related with ulcer and inflammation.
